# Supplementary material for: Behavioural diversity of bonobo prey preference as a potential cultural trait
Source: eLife. 2020 Sep 1;9:e59191. doi: 10.7554/eLife.59191 (PMC7462605; doi:10.7554/eLife.59191)
Supplement: Supplementary file 1. — The identity and sex (M = male, F = Female) of the individual that caught the prey is noted whenever information was available (NA = unknown). The party composition columns depict the different group members that were present during the hunt scan. In bold presented are the individuals that participated in the hunt (i.e., chased prey) when known. Note, we have at times likely underestimated the number of hunters. [file elife-59191-supp1.docx]

**Supplementary File 1**. Successful hunt cases on anomalure, duiker, and squirrel species documented between August 2016 and January 2020 in Ekalakala (EKK) and Kokoalongo (KKL). The identity and sex (M = male, F = Female) of the individual that caught the prey is noted whenever information was available (NA = unknown). The party composition columns depict the different group members that were present during the hunt scan. In bold presented are the **individuals that participated in the hunt** (i.e., chased prey) when known. Note, we have at times likely underestimated the number of hunters.

| Prey Species | Party composition EKK | Party composition KKL | Capture | |
| --- | --- | --- | --- | --- |
|  |  |  | Subject | Sex |
| Anomalure | Azur, Bleue, Eben, Gris, Ivoire, Noir, Peche, Rouge, Violette |  | Azur | F |
|  | Azur, Bleue, Eben, Ivoire, Noir, Peche, Rouge, Violette |  | Bleue | F |
|  | Azur, Bleue, Eben, Gris, Ivoire, Noir, Peche, Rouge, Violette |  | Bleue | F |
|  | Bleue, **Eben**, Noir, **Olive**, Violette |  | Eben | F |
|  | Azur, Bleue, **Gris**, **Ivoire**, Noir, Rouge, **Violette** | Adele, Bowie, Dion, Eliot, Fitz, Hendrix, Izia, Jackson, Kidjo, Papa Wemba, PJ, Sting, Tyler | Ivoire | F |
|  | **Azur**, **Bleue**, Eben, Gris, **Ivoire**, **Noir**, **Peche**, Rouge, Violette |  | Ivoire | F |
|  | Azur, **Bleue**, Eben, Gris, Ivoire, **Noir**, Peche |  | Noir | M |
|  | Azur, Bleue, Eben, Gris, **Ivoire**, **Noir**, **Peche**, Violette |  | Noir | M |
|  | Azur, Eben, **Olive, Peche, Rose, Violette** |  | Rose | F |
|  | Bleue, Eben, Rouge, Violette |  | Rouge | M |
|  | Bleue, **Eben**, **Ivoire**, Olive, Peche, **Rouge**, **Violette** |  | Violette | F |
|  | **Bleue**, **Eben**, Olive, Peche, Rose, **Violette** |  | Violette | F |
|  | Bleue, Gris, Ivoire, Noir, Olive, Rouge, Violette |  | Violette | F |
|  | Azur, Bleue, Eben, Gris, Ivoire, Noir, Peche, Rouge, Violette |  | NA |  |
|  | Azur, Bleue, Eben, Gris, Ivoire, Noir, Peche, Rouge, Violette |  | NA |  |
|  | Azur, Bleue, Eben, Ivoire, Noir, Peche, Rouge, Violette |  | NA |  |
|  | Bleue, Eben, Ivoire, Noir, Rouge, Violette |  | NA |  |
|  | Azur, Bleue, Eben, Gris, Ivoire, Noir |  | NA |  |
|  | Azur, Bleue, Peche, Rouge, Violette |  | NA |  |
|  | Azur, Bleue, Ivoire, Noir, Violette |  | NA |  |
|  | Azur, Bleue, Eben, Ivoire, Noir, Peche, Violette |  | NA |  |
|  | Azur, Bleue, Eben, Gris, Ivoire, Noir, Peche, Rouge, Violette |  | NA |  |
|  | Azur, Bleue, Ivoire, Peche, Rouge, Violette |  | NA |  |
|  | Azur, Bleue, Eben, Ivoire, Noir, Peche, Rouge, Violette |  | NA |  |
|  | Azur, Bleue, Eben, Gris, Ivoire, Noir, Violette |  | NA |  |
|  | Azur, Bleue, Eben, Ivoire, Noir, Peche, Rouge, Violette |  | NA |  |
|  | Azur, Bleue, Eben, Gris, Noir, Peche, Rouge |  | NA |  |
|  | Azur, Bleue, Gris, Noir |  | NA |  |
|  | Azur, Bleue, Eben, Gris, Noir, Peche, Violette |  | NA |  |
|  | Bleue, Eben, Ivoire, Olive, Peche, Violette |  | NA |  |
|  | Azur, Bleue, Eben, Ivoire, Noir, Olive, Peche, Rouge, Violette |  | NA |  |
|  |  | **Chapman, Hendrix**, Oliday, PJ, Presley, **Tyler** | Chapman | F |
|  |  | Adele, Fito, **Fitz**, Papa Wemba | Fitz | F |
|  | Azur, **Noir, Eben, Violette** | Chapman, Nico, **Oliday**, PJ | Oliday | F |
|  |  |  |  |  |
| Duiker | **Bleue**, Eben, Ivoire, Noir, Peche, Rose, Rouge, **Violette** |  | Bleue | F |
|  |  | Chapman, Eliot, Gloria, Madonna, Marley, Papa Wemba, Ray, Simone, Zappa | Chapman | F |
|  | Eben | Adele, **Fitz**, Jackson, **Kidjo**, Madonna, Ray | Fitz | F |
|  | **^1^** | Fitz, Kidjo, Love, Madonna, Papa Wemba, Zappa | Fitz | F |
|  |  | Eliot, **Gloria**, Hendrix, Oliday, Zappa | Gloria | F |
|  | Eben, Gris, Ivoire, Noir, Rouge | Adele, Chapman, Dion, **Eliot**, Fito, Fitz, **Gloria**, Hendrix, Izia, Jackson, Kidjo, **Nico**, **Oliday**, PJ, Presley, Ray, Simone, Tyler, Wonder, Zappa | Oliday | F |
|  | Azur, Ivoire | Adele, Bowie, Chapman, Dion, Eliot, Fito, Fitz, Gloria, Izia, Madonna, Marley, Nico, Oliday, **Papa Wemba**, Presley, Simone, Tyler, Wonder, Zappa | Papa Wemba | M |
|  |  | Bowie, Chapman, Eliot, Fitz, Gloria, Hendrix, Jackson, Kidjo, Oliday, Papa Wemba, Presley, Simone, Tyler | Papa Wemba | M |
|  |  | **Chapman**, Eliot, Gloria, Izia, Nico, PJ, Presley, Ray, Simone, **Zappa** | Zappa | M |
|  |  | Adele, Bowie, Fito, Fitz, Kidjo, Love, Madonna, Marley, Sting | NA |  |
|  | **^2^** | Chapman, Dion, Eliot, Fitz, Gloria, Nico, Oliday, PJ, Tyler, Zappa | NA |  |
|  |  | Gloria, Simone, Tyler | NA |  |
| **^1^** Ivoire was traveling with Kokoalongo in the immediate scan before the hunt  **^2^** Bleue was traveling with Kokoalongo in the immediate scan before the hunt and re-joined the party after the duiker was captured | | | | |
| Squirrel | Azur, Bleue, Eben, Gris, Ivoire, Noir, Rouge, Violette |  | NA |  |
|  |  | Chapman, Dion, Jackson, Kidjo, Papa Wemba, Ray, Tyler | Chapman | F |
|  |  | Bowie, **Chapman**, Dion, Eliot, Fitz, Gloria, Hendrix, Jackson, Kidjo, Madonna, Oliday, PJ, Simone, Zappa | Chapman | F |
|  | Eben, Gris, Ivoire, Noir | Chapman, Dion, Eliot, Gloria, Hendrix, Izia, Nico, PJ, Ray, Simone, Tyler, Wonder, Zappa | Chapman | F |
|  |  | Adele, Bowie, Chapman, Eliot, Fito, Fitz, Jackson, Kidjo, Madonna, Oliday, PJ, Presley, Simone, Zappa | Fitz | F |
|  |  | Chapman, Dion, Eliot, Gloria, Hendrix, Tyler | Gloria | F |
|  |  | Oliday | Oliday | F |
|  |  | Chapman, Eliot, PJ, Presley, Simone, Zappa | PJ | F |
|  |  | Chapman, PJ, Presley | Presley | M |
|  |  | Chapman, Dion, Eliot, Izia, PJ, Presley, Ray, Simone, Tyler | Simone | F |
|  |  | Dion, Gloria, Jackson, Papa Wemba, Ray, Simone, Tyler, Zappa | Zappa | M |
|  |  | Chapman, Eliot, PJ, Presley, Simone | NA |  |
|  |  | Chapman, Eliot, Gloria, Oliday, Ray, Simone | NA |  |
