# Supplementary material for: Behavioural diversity of bonobo prey preference as a potential cultural trait
Source: eLife. 2020 Sep 1;9:e59191. doi: 10.7554/eLife.59191 (PMC7462605; doi:10.7554/eLife.59191)
Supplement: Supplementary file 2. [file elife-59191-supp2.docx]

**Supplementary File 2**. Demography of the adult individuals of the Ekalakala and Kokoalongo groups, 2016-2020 (M = male, F = Female).

| Subject | Group | Sex | Age (yrs) |
| --- | --- | --- | --- |
| Azur | Ekalakala | F | 15-25 |
| Bleue |  | F | >35 |
| Eben |  | F | 10-15 |
| Gris |  | M | 15-25 |
| Ivoire |  | F | 25-35 |
| Peche |  | F | 15-25 |
| Noir |  | M | 15-25 |
| Olive |  | F | 10-15 |
| Rose |  | F | 10-15 |
| Rouge |  | M | >35 |
| Violette |  | F | 25-35 |
| Adele | Kokoalongo | F | 25-35 |
| Bowie |  | M | 15-25 |
| Chapman |  | F | 25-35 |
| Dion |  | F | >35 |
| Elliot |  | F | 15-25 |
| Fito |  | M | 10-15 |
| Fitz |  | F | 25-35 |
| Gloria |  | F | 25-35 |
| Hendrix |  | M | 15-25 |
| Izia |  | F | 25-35 |
| Jackson |  | M | >35 |
| Kidjo |  | F | 15-25 |
| Love |  | F | >35 |
| Madonna |  | F | >35 |
| Marley |  | M | 25-35 |
| Oliday |  | F | 15-25 |
| PapaWemba |  | M | 15-25 |
| PJ |  | F | 15-25 |
| Presley |  | M | 10-15 |
| Nico |  | F | 10-15 |
| Ray |  | M | 25-35 |
| Simone |  | F | >35 |
| Sting |  | M | >35 |
| Tyler |  | F | 25-35 |
| Zappa |  | M | 10-15 |
| Wonder |  | M | 15-25 |
